# Anticholinergic Drugs in Geriatric Psychopharmacology

**DOI:** 10.3389/fnins.2019.01309

**Published:** 2019-12-06

**Authors:** Jorge López-Álvarez, Julia Sevilla-Llewellyn-Jones, Luis Agüera-Ortiz

**Affiliations:** ^1^Servicio de Psiquiatría, Hospital 12 de Octubre (imas12), Madrid, Spain; ^2^Instituto de Investigación, Hospital 12 de Octubre (imas12), Madrid, Spain; ^3^Instituto de Psiquiatría y Salud Mental, Hospital Clínico San Carlos, Madrid, Spain; ^4^Instituto de Investigación (IdISSC), Hospital Clínico San Carlos, Madrid, Spain; ^5^Centro de Investigación Biomédica en Red de Salud Mental, Ministry of Science and Innovation, Madrid, Spain; ^6^Departamento de Medicina Legal, Psiquiatría y Patología, Facultad de Medicina, Universidad Complutense de Madrid, Madrid, Spain

**Keywords:** anticholinergic drugs, elderly, potentially inappropriate prescriptions, adverse effects, cognition, dementia

## Abstract

Drugs with anticholinergic action are widely prescribed in the elderly population due to their potential clinical benefits. However, these benefits are limited by adverse effects which may be serious in particular circumstances. This review presents different aspects of the use of anticholinergics in old age with a focus in psychogeriatric patients. We critically review published data on benefits and disadvantages of anticholinergics, which are often controversial. Prevalence, pathophysiology and measurement methods of the anticholinergic action of drugs are discussed. We also present the most important drawbacks resulting from its use, including effects on cognition in healthy and cognitively impaired people, in aged schizophrenia patients, emergence of delirium and psychiatric symptoms, influence in functionality, hospitalization, institutionalization and mortality, and the potential benefits and limitations of their discontinuation. Finally, we suggest practical recommendations for the safe use of anticholinergics in clinical conditions affecting elderly patients, such as dementia, schizophrenia and acute hallucinatory episodes, depression, anxiety, Parkinson’s disease, cardiovascular conditions and urinary incontinence.

## Introduction

Anticholinergic drugs bind to the muscarinic receptors and block acetylcholine neurotransmission, which is involved in many major body functions including central nervous system (CNS) functions such as attention, learning and memory mechanisms and peripheral nervous system (PNS) actions which are related to basal functioning of the organism such as urination, intestinal transit or heart rhythm regulation. Drugs with anticholinergic action may bind exclusively to muscarinic receptors or may also bind to other receptors with agonist or antagonist actions, and therefore, have different therapeutic targets and denominations (e.g., tricyclic antidepressants).

Since cholinergic transmission is involved in many physiological functions, anticholinergic drugs can have adverse effects and affect the central and peripheral nervous systems. On the one hand, adverse effects on the central nervous system (CNS) include dysfunction in different cognitive domains, cognitive impairment, an acceleration of neurodegenerative processes, the appearance of psychotic or confusional symptoms and functionality disturbances. On the other hand, adverse effects on the PNS include dry mouth, urinary retention, constipation and paralytic ileus, increased heart rate and blurred vision, among others.

## Anticholinergic Drugs

Anticholinergic drugs are used for multiple medical conditions, such as urinary dysfunction, peptic ulcer disease, irritable bowel syndrome or Parkinson’s disease. Moreover, anticholinergics are frequently used as anesthetic agents, and for neurologic and psychiatric conditions (Potamianos and Kellett, 1982). While some anticholinergic drugs are well known and their anticholinergic effects are fully recognized, other drugs have less known anticholinergic activity (AA). Table 1 shows the most frequently used neuropsychiatric drugs with anticholinergic effects and Table 2 some commonly prescribed non-psychoactive drugs with anticholinergic effects.

**TABLE 1:** Neuropsychiatric drugs with anticholinergic side effects.

**High anticholinergic load**
**Tryciclicantidepressants**
Amitryptiline
Clomipramine
Desipramine
Imipramine
**Antipsychotics**
Chlorpromazine
Clozapine
Fluphenazine
Loxapine
Olanzapine
Perphenazine
**Antiepileptics**
Carbamazepine
Oxcarbazepine
**Anticholinergics as such**
Benztropine
Biperiden
Trihexyphenidyl
**Low-moderate anticholinergic load**
**Antidepressants**
Fluoxetine
Fluvoxamine
Mirtazapine
Nortriptiline
Paroxetine
Sertraline
Trazodone
**Antipsychotics**
Haloperidol
Quetiapine
Ziprasidone
**Benzodiazepines**
Alprazolam
Chlordiazepoxide
Clonazepam Clorazepate
Diazepam
Flurazepam
Lorazepam
Midazolam
**Antiepileptics**
Valproate
**Antiparkinsonians**
Amantadine
Bromocriptine
Carbidopa- Levodopa
Pramipexol
Selegiline
**Opioids**
Codeine
Fentanyl
Morphine
Tramadol

**TABLE 2:** Common non-psychoactive drugs with anticholinergic effects.

**Antihistamines**
Diphenhydramine
Hydroxyzine
Loratadine
**Antispasmodics**
Loperamide
**Antiulcer**
Cimetidine
Ranitidine
**Bronchodilatadors**
Fluticasone- salmeterol
**Cardiovascular**
Digoxine
Diltiazem
Warfarin
**Corticoids**
Cortisone
Dexamethasone
Methylprednisolone
Prednisone
**Diuretics**
Captopril
Chlortalidone
Furosemide
**Urologycal**
Oxybutynin
Tolterodine

The main properties of anticholinergic drugs are related to their action on central and/or peripheral cholinergic receptors (Lechevallier-Michel et al., 2005). These actions can cause symptoms such as cognitive disorders, which can be mistakenly assumed as part of normal manifestations of aging (Kersten et al., 2013).

It is also important to note that some drugs, such as amoxicillin, diazepam, digoxin, duloxetine, fentanyl, furosemide, lansoprazole, metformin, phenytoin or topiramate only have AA at high doses (Chew et al., 2008). Furthermore, the combination of different drugs (or even nutritional supplements such as chondroitin or some vitamins) can cause or exacerbate the adverse events of an already prescribed anticholinergic drug.

Anticholinergics have many side effects and depend on the anticholinergic drug load and individual vulnerability. Table 3 summarizes the side effects of anticholinergic agents attending to the anticholinergic load.

**TABLE 3:** Anticholinergic side effects of drugs.

**Mild or moderate anticholinergic effects**	**Severe anticholinergic effects**
**Somatic symptoms**	**Somatic symptoms**
Anhidrosis	Congestive heart failure
Blurry vision	Fecal impaction/paralytic ileus
Constipation	Malnutrition
Dry mouth	Respiratory infections
Fatigue	Tachyarrhythmia
Mydriasis	Urinary retention/urinary tract infection
Tachycardia/palpitations	Cardiac attack
Urinary hesitancy	
**Neuropsychiatric symptoms**	**Neuropsychiatric symptoms**
Drowsiness	Agitation
Nervousness, excitement	Ataxia
Mild amnesia and cognitive dysfunction	Complex visual hallucinations
Poor attention	Delirium
Restlessness	Epileptic seizures
	Hallucinations
	Hyperreflexia
	Nocturnal rhythm disturbance

Due to their side effects, the prescription of anticholinergic drugs may be considered inappropriate in certain circumstances. Potentially inadequate anticholinergic drug use in older adults is included in the Beers (American Geriatrics Society 2012 Beers Criteria Update Expert Panel, 2012) and the STOPP-START criteria (O’Mahony et al., 2015). However, for certain clinical syndromes the benefits of anticholinergics are greater than their risks, and their prescription can be considered adequate in the elderly in some cases (Table 4).

**TABLE 4:** Clinical problems in the elderly population where the prescription of anticholinergics may be appropriate or inevitable in certain circumstances.

**Psychiatric conditions**	**Non-psychiatric conditions**
Primary psychotic disorders (schizophrenia, schizoaffective disorder, delusional disorder)	Gastroesophageal reflux disease
Bipolar disorder	Cardiovascular diseases
Endogenous depression	Irritable bowel syndrome
Psychotic depression	Muscle spasms/low back pain
Obsessive-Compulsive disorder	Neuropathic pain
Severe anxiety disorders	Urinary incontinence (after non-pharmacological approach)
Severe insomnia	
Drug-induced acute dystonia	
Secondary parkinsonism	

## Pathophysiology of Anticholinergic Drugs in the Brain

There are five subtypes of muscarinic receptors, M1 to M5. M1, M2 and M4 are, to date, muscarinic receptors that appear to be more related to the possible anticholinergic deleterious effects. M1 receptors are the most common in the CNS and have a crucial role related to executive functions and episodic memory in the prefrontal cortex and hippocampus, respectively (Sathienluckana et al., 2018). M2 receptors participate in memory processing and M4 receptors regulate acetylcholine levels. The antagonism of these receptors can lead to cognitive disturbance and cell death (Bishara et al., 2017). In fact, alteration of cholinergic function affects gating of sensory input and information processing, prerequisites for learning and memory. Cognitive effects depend on anticholinergic load, baseline cognitive function and individual pharmacokinetics and pharmacodynamics variability, and are also influenced by metabolism alterations (Roe et al., 2002).

Anticholinergic drugs only produce central effects if crossing the blood-brain barrier. This is less likely if the drug is a substrate of the *P*-glycoprotein multi-drug efflux pump (Bishara et al., 2017) and more likely if the activity of that transporter is reduced. In this line, it is important to note that blood-brain barrier permeability is increased in people with diabetes or multiple sclerosis, and by drugs such as lansoprazole, omeprazole, loperamide, simvastatin, clonidine, or methyldopa.

Long-term continuing therapy with anticholinergic drugs could induce brain changes partially comparable to those present in Alzheimer’s disease. In fact, cognitive dysfunction in AD is related with different aspects, such as decrease in the number of cholinergic neurons, acetylcholine receptor dysfunctions, and signaling dysregulation. Decline severity is proportional to the alteration of the cholinergic system (Swami et al., 2016). In this line, an anatomopathological study found that amyloid plaques were 2.5 times more frequent and neurofibrillary tangles were increased in patients with Parkinson’s disease treated with anticholinergic drugs in the long term compared to short-term treated or non-treated patients (Perry et al., 2003). However, these findings were not confirmed in a similar study (Gray et al., 2018). Interestingly, Caccamo et al. (2006) found that long-term cholinergic receptor blockade increased the beta-amyloid peptide in the cortex, hippocampus and amygdala.

Using neuroimaging techniques, a cross-sectional study in older adults found an association between moderate or high anticholinergic load and reduced cerebral metabolism, decreased cortical volume and thickness of the temporal cortex, increased ventricular volume, temporal lobe atrophy and global atrophy (Risacher et al., 2016). Accordingly, a longitudinal study detected higher rates of atrophy in the total cerebral volume and in the gray matter associated to anticholinergic drug consumption, finding a dose-dependent relationship between anticholinergic load and cortical thinning (Chuang et al., 2017). A different study linked the integrity of the cholinergic system with the correct functioning of episodic memory (Richter et al., 2014). Additionally, Sperling et al. (2002) found that the administration of scopolamine, a potent anticholinergic drug, decreased brain activation in the hippocampus, fusiform gyrus and lower frontal regions, and was associated with lower performance in neuropsychological tests.

The concept of “anticholinergic spectrum disorders” has been proposed by Hori et al. (2016), which would include Alzheimer’s disease, Lewy body dementia and delirium. They consider that acetylcholine downregulation causes an endogenous AA cascade that can exacerbate neurodegeneration (Kitajima et al., 2015). This symptomatology can be precipitated by a moderate and asymptomatic cholinergic deficit in the presence of “AA inserts” such as polypharmacy, physical illnesses or stress, and is reversible when the action of these “AA inserts” disappears. However, when endogenous AA appears, irreversible progression to dementia occurs (Konishi et al., 2015). In relation to this, mice experiments showed that a greater anticholinergic action increased tau pathology and its insolubility, which could be reversed with the use of procholinergic drugs (Yoshiyama et al., 2015). In fact, acetylcholinesterase inhibitors (AchI) increase cholinergic activity, and although they do not halt disease progression, they are currently considered the treatment of choice for dementias presenting with cholinergic deficit, such as Alzheimer’s disease, Lewy body dementia or Parkinson’s dementia.

Additionally, psychotic symptoms in Alzheimer’s disease have been explained by a central cholinergic deficiency syndrome that would cause dysfunction in detecting, selecting, discriminating and processing sensory inputs (Lemstra et al., 2003). Thus, psychosis is associated with metabolic and perfusion alterations in the temporal and frontal cortices, where the cholinergic deficit is greater (Cummings and Back, 1998). Psychotic symptoms in Alzheimer’s disease are also associated with elevated binding to M2 receptors at frontal and temporal cortical level (Lai et al., 2001).

## Prevalence

Despite the deleterious effects associated to the use of anticholinergic drugs in the elderly, these medications are widely prescribed, mainly because they are efficacious and less harmful alternatives are not available. For example, in Primary Care, a prevalence of 12.5% of prescriptions was detected in this population. Among these, more than half were for a psychotropic drug (Gorup et al., 2018). In fact, the prevalence increases to 25.8% if the aged person presents with mnesic complaints (Grande et al., 2017).

Anticholinergic drugs consumption has been associated with lower educational level, chronic comorbidities (Grande et al., 2017), hospitalization or specialist visits in the previous year, urinary incontinence, arterial hypertension (Niznik et al., 2017), impaired health status, anxiety, mood disorders (Kachru et al., 2015) and polypharmacy.

While it has been reported that during hospital admission the number of patients who receive anticholinergics and their related anticholinergic load increases, it is not yet clarified whether this is related to the medical circumstances of the hospitalization or the circumstances around the hospitalization itself (Brombo et al., 2018). However, this increase affects 79% of the hospitalized patients (Pasina et al., 2019). In addition, around the 20% of the patients in specialized geriatric hospitalization units -a setting where the use of anticholinergics is often well scrutinized -receive high-potency drugs (Pfistermeister et al., 2017), which supports the idea that in a significant number of cases, this use is not easily avoidable.

In nursing homes, the most prevalent anticholinergics were cardiovascular drugs, followed by antipsychotics and antidepressants (Wu et al., 2017). These findings are in line with those of a community-dwelling elderly patients study (Green et al., 2016).

In patients with mild cognitive impairment (MCI) or dementia treated in memory clinics, 44.7% were taking anticholinergic drugs, with 11.7% receiving a high anticholinergic load (Cross et al., 2016). Up to half of the patients with MCI have a high anticholinergic load due to the additive effect of several drugs (Green et al., 2016). The prevalence of anticholinergic use is increased in the institutionalized elderly, especially if they are diagnosed with dementia, and even in those with a terminal condition (Williams et al., 2018).

In earlier clinical studies the potentially inappropriate prescription of anticholinergics was not usually taken into consideration (Kachru et al., 2015). Nowadays, the Beers (American Geriatrics Society 2012 Beers Criteria Update Expert Panel, 2012) and the STOPP-START criteria (O’Mahony et al., 2015) consider that their prescription is inappropriate in most cases. However, whether or not prescribing anticholinergics is appropriate is something still unclear (López-Álvarez and Agüera Ortiz, 2019). In fact, a study using the Beers criteria found that 39.9% of elderly people with dementia on an outpatient basis were taking anticholinergic drugs classified as potentially inadequate (Bala et al., 2019).

Despite their antagonistic effects, the combined use of AchI and anticholinergics is common, especially when depression, chronic obstructive pulmonary disease, hypertension, chronic kidney disease and/or cardiac failure are present (Mantri et al., 2019). This combination is frequently used to treat procholinergic side effects, such as nausea, dizziness, diarrhea or incontinence (Roe et al., 2002), even when a non-anticholinergic suitable alternative is available (Carnahan et al., 2004). Several investigations suggest the existence of a vicious cycle of prescription of anticholinergics and procholinergics. In fact, Gadzhanova et al. (2015) described that around 1/3 of the patients who receive AchI or memantine have received anticholinergic drugs in the previous months. They also found that 12% of *de novo* prescriptions of these drugs were done after the start of anti-dementia drugs. Different “combined use” prevalence rates have been found depending on the detection tool, the clinical context and the baseline disorder. For example, prevalence varies from 44.9% (with 11.3% taking a high anticholinergic load) (Cross et al., 2016), or 54.1% in hospitalized elderly (Weichert et al., 2018), to 81.3% in patients with Parkinson dementia (with 44.5% taking a high anticholinergic load) (Mantri et al., 2019).

## Measurement of Anticholinergic Action

Anticholinergic action cannot be directly measured. Traditionally, anticholinergic load has been inferred through the use of laboratory techniques or scales and lists of anticholinergic drugs.

### Lab Techniques

The serum anticholinergic activity (SAA) is the most frequently used laboratory technique and has been considered the gold standard method for measuring anticholinergic action for years (Tune and Coyle, 1980). It measures the peripheral anticholinergic action, thus not reflecting the central action. Under baseline conditions no anticholinergic activity is detected in serum, although it might be detected in the absence of drugs with anticholinergic action, due to a non-specific reaction to stress or the use of polypharmacy (Flacker and Lipsitz, 1999; Plaschke et al., 2010). In addition, it does not differentiate the agonistic or antagonistic activity of the muscarinic receptor (Collamati et al., 2016) and it does not assess the effects of metabolites such as *N*-desmethylclozapine. The combination of anticholinergic drugs of different potency can increase or decrease the anticholinergic action of the drug with greater anticholinergic action, which could also happen if an anticholinergic drug is combined with a pro-cholinergic drug (Mayer et al., 2016). Other limitations to its clinical use are cost and availability.

Alternative laboratory techniques are being studied, such as blood acetylcholinesterase and blood butyrylcholinesterase levels. These techniques have shown high sensitivity in detecting drugs with anticholinergic action, but not in assessing synergistic effects of different anticholinergic agents (Plaschke et al., 2016).

### Scales and Indexes of Anticholinergic Drugs

Drug scales are objective, reproducible and easy-to-administer tools to determine anticholinergic action. They can be applied in a short time, especially when online calculators are used. Many of them are based on SAA published data, so they present the limitations already described. In addition, they have other limitations such as not considering pharmacological interactions, not being homogeneous in terms of anticholinergic load attribution (e.g., in the case of quetiapine) (Brombo et al., 2018), and not usually considering the daily dose, receptor affinity, blood-brain barrier permeability, serum or tissue concentrations (Ruxton et al., 2015), renal and hepatic functions, comorbidities, and the use of other drugs. As additional limitations, they may also be outdated, and thus, underestimate the anticholinergic load, or be ambiguous because they do not indicate if there is a lack of AA or simply this has not been assessed (Bishara et al., 2017). The obtained results are not completely translatable worldwide, since these scales may include drugs that are not commercialized or approved in a specific country. In addition to all these limitations, it must be pointed out that these scales tend to assume that the anticholinergic load is linear and additive when it is unlikely that it is proportional to a ratio of 0: 1: 2: 3 (Salahudeen et al., 2015). Furthermore, considering the finite number of muscarinic receptors, a plateau effect can be reached at some point, which in fact implies that the sum of the anticholinergic effects of the individual drugs may overestimate the total anticholinergic load. Despite all these limitations, scales seem more useful than *in vitro* determinations to facilitate clinical decisions (Wawruch et al., 2012).

The scales more frequently used are the following:

• Anticholinergic Drug Scale (ADS). It classifies drugs according to their AA. Scores varies from 0, for lack of activity; 1, if a possible anticholinergic action exists, to 2–3 for high activity. The SAA, the pharmacological characteristics of each drug and the clinical experience were considered for its design. It includes a large number of medications, including non-oral formulations (Carnahan et al., 2006).
• Anticholinergic Risk Scale (ARS). Its scoring system is similar to the ADS scale, ranging from 0 to 3 points. It is based on the dissociation constant of the muscarinic receptor, the rates of anticholinergic effects and a literature review of about 500 drugs. It includes a smaller number of drugs, mainly psychotropics, and excludes non-oral formulations (Rudolph et al., 2008).
• Anticholinergic Cognitive Burden Scale (ACB). In this scale, drugs are classified according to SAA or *in vitro* affinity to muscarinic receptors. Drugs with ability to produce SAA or to bind muscarinic receptors *in vitro* score 1, even if no relevant cognitive effects are shown. Drugs score 2 when some cognitive effects can be found and 3 when these cognitive effects are very significant. This scale includes a number of medicines similar to the ADS scale and is the scale most commonly used in research studies (Boustani et al., 2008).
• Drug Burden Index (DBI). It is not a scale, but a summation of the anticholinergic load, the sedative load or the combination of the anticholinergic load and the sedative load. This index is calculated by the ratio between the prescribed dose and the sum of the minimum dose and the prescribed dose. The greater the difference between the minimum dose and the prescribed dose, the greater the individual load for that drug (Hilmer et al., 2007). Its values range from 0 to ∞. Besides oral formulations, it also includes topical or inhaled drugs. As an advantage, it considers the drug dose, although it does not differentiate between mild or potent anticholinergics (Wouters et al., 2017). In a systematic review it was considered the best tool for the longitudinal evaluation of anticholinergic effects (Cardwell et al., 2015).
• Other tools used to measure anticholinergic action include the Han et al. (2001), Chew et al. (2008), and Durán et al. (2013) lists, as well as the following scales: Anticholinergic Burden Classification Scale (ABC) (Ancelin et al., 2006), Anticholinergic Activity Scale (AAS) (Ehrt et al., 2010), Anticholinergic Loading Scale (ACL) (Sittironnarit et al., 2011) and Anticholinergic Impregnation Scale (AIS) (Briet et al., 2017).

These different scales have been compared in several studies. One study showed that only 29 drugs were present in all the main scales used (ACB, ARS, and ADS) and only 20 if DBI was included (Naples et al., 2015). These scales included a wide number of drugs, and the correlations among them are usually low. Therefore, results among scales are different and not interchangeable (Brombo et al., 2018). ADS and ACB have an adequate correlation as their design is based on similar data, and are the scales that detect more anticholinergic drugs (Pont et al., 2015). It has been suggested that the ARS scale is better for the detection of cognitive impairment than the ACB scale, whereas the ACB is better to assess functionality, although it has not been clearly established that some tools are more specific than others for assessing specific side effects (Pasina et al., 2013).

In summary, there are several techniques to indirectly assess anticholinergic activity of drugs. The gold standard is the determination of SAA. However, this technique is not usually accessible and has some limitations. Several rating scales for anticholinergic drugs have been developed, a procedure that is more accessible, but also limited. The correlation between these scales is not high and there is little evidence of superiority of one over the other either in general or to detect specific outcomes.

## Outcomes

Different outcomes have been associated to the intake of anticholinergic drugs, beyond cognitive disturbance. However, when studying this topic, it is important to consider reverse-causation bias, i.e., that the medical condition itself, and not the anticholinergic drug used to treat it, causes the outcome. This limitation is normally solved in longitudinal studies. However, longitudinal studies are scarce since they are difficult and expensive to perform. Another difficulty is that drugs with anticholinergic action may bind to different receptors other than the muscarinic receptor which may increase or avoid certain side effects related to the anticholinergic effect (Ruxton et al., 2015).

### Cognitive Outcomes in Healthy Subjects

Adverse cognitive effects of anticholinergic drugs have been evaluated in cross-sectional and longitudinal studies in healthy subjects, in subjects with MCI and in subjects with dementia. However, there is no unanimous evidence of the cognitive effects of anticholinergic drugs and whether these effects are reversible or not (Salahudeen and Nishtala, 2016). The studies in which no association was found have been questioned by a possible protopathic bias or reverse causation due to the greater use of these drugs to treat dementia prodrome symptoms. Another artifact could be the use of the Mini-Mental State Examination (MMSE), the most widely used cognitive screening test, which is now being questioned as an adequate tool to detect subtle changes induced by drugs (Salahudeen et al., 2016). In this line, more extensive neuropsychological batteries are now recommended instead (Wouters et al., 2017). Regarding the heterogeneity of the results, another hypothesis suggests that it may be due to the heterogeneity of the cholinergic reserves of the participants (Collamati et al., 2016) which could explain the lack of association between cognition and anticholinergic load in middle-aged persons (Limback-Stokin et al., 2018).

Already in 1973 it was suggested that blocking muscarinic receptors altered learning processes (Crow and Grove-White, 1973). Subsequent cross-sectional studies supported an association between anticholinergic load and different cognitive parameters, such as episodic memory, executive functions and psychomotor speed (Ziad et al., 2018), recall of a short story and a list of words (Potamianos and Kellett, 1982), visual immediate memory and verbal fluency (Lechevallier-Michel et al., 2005), immediate memory and delayed memory (Nakra et al., 1992), memory and registry domains of the MMSE (Konishi et al., 2010), color and interference in the Stroop test (Sittironnarit et al., 2011) and free immediate memory, total immediate memory, free delayed memory and total delayed memory (Fortin et al., 2011).

Despite the importance of cross-sectional studies, it has been suggested that for some cognitive domains, dysfunction could only be proved in longitudinal studies (Gnjidic et al., 2012). In addition, these studies can assess if anticholinergic drugs increase the risk of dementia or produce a reversible cognitive dysfunction. It has also been suggested that even an initially asymptomatic anticholinergic exposure can cause cognitive dysfunction in the long term after a prolonged used of anticholinergic drugs (Han et al., 2008). In longitudinal studies, anticholinergic action can accelerate the decline of the components of executive function such as attention, sequencing, concentration and cognitive flexibility (Bottiggi et al., 2006).

Intake of anticholinergics has been associated with a greater decline of MMSE scores (Fox et al., 2011b) regardless of functional decline (Brombo et al., 2018). A brief exposure to drugs with anticholinergic action is associated with cognitive impairment, but not with dementia(Ancelin et al., 2006; Jamsen et al., 2017). In contrast, a prolonged exposure has been associated with dementia and not only with cognitive impairment (Cai et al., 2013). The association between long-term exposure and dementia suggests that the time factor and/or the cumulative dose influence the development of a neurodegenerative process. However, it is currently unknown whether the time exposure factor is a necessary or sufficient condition to increase the risk of dementia. Thus, Whalley et al. (2012) showed that previous cognitive impairment is more frequent in patients who took anticholinergics.

In relation to the transition from cognitive normality to MCI, Campbell et al. (2018) suggested that the continued use of potent anticholinergics may increase conversion rates. In contrast, Chuang et al. (2017) argued that the continued use and not the high anticholinergic load is related to incident MCI and dementia, as high potency anticholinergics are usually prescribed only for short periods of time. In this line, other works have also found a higher risk of incident dementia (Carrière et al., 2009), finding even a dose-dependent relationship (Jessen et al., 2010; Gray et al., 2015; Richardson et al., 2018). Some authors have suggested that the exposure to potent anticholinergics and the risk of dementia or Alzheimer’s disease is independent of the time elapsed from such exposure to the moment of diagnosis (Gray et al., 2015). Nevertheless, the relationship between the intermittent use of anticholinergic drugs and cognitive effects in the medium and long term is not yet sufficiently clear (Araklitis et al., 2017).

The prolonged use of medications with anticholinergic activity such as antidepressants, urological and antiparkinsonian drugs has been associated with increased risk of dementia (Richardson et al., 2018). In the case of antidepressants, reverse causation is suspected if the drug has not been used for a long time.

The presence of the APOE 4 allele is widely recognized as a risk factor of dementia. However, the results about the influence of APOE 4 allele on cognitive dysfunction mediated by anticholinergic drugs are contradictory. While cross-sectional studies support E4 allele to be associated with poorer cognitive performance (Pomara et al., 2004; Uusvaara et al., 2009), longitudinal studies either found no association (Gray et al., 2015) or deterioration in MMSE scores only in females (Carrière et al., 2009).

Summing-up the information related to cognitively normal persons, the self-limited anticholinergic action on cognition in this population is well known long ago. It requires the interaction between the previous cognitive level and the potency of the anticholinergic action. However, while some longitudinal studies have found a relationship between the continued use of anticholinergic drugs and the development of Alzheimer’s pathophysiology, others have only found a reversible cognitive impairment. Therefore, it is suggested that the exposure time or the cumulative dose of anticholinergics may end up exceeding a threshold from which the neurodegenerative process is triggered and the anticholinergic effect is not reversible anymore.

### Cognitive Outcomes in Previously Cognitive Impaired Patients

The anticholinergic load may be a modifiable risk factor for dementia (Green et al., 2016). It is estimated that between 2 and 12% of patients with suspected dementia actually have pharmacological iatrogenesis in that respect (Moore and O’Keeffe, 1999). Patients with SAA levels at a 90th percentile or higher were 13 times more likely to have a MMSE of 24 or lower compared to patients without detectable SAA (Mulsant et al., 2003). In addition, a greater progression to MCI or dementia has also been found if both anticholinergic load and positivity to beta-amyloid are present (Risacher et al., 2016).

In a sample of primary care patients with first cognitive complaints, an elevated anticholinergic load was required for a significant association with cognitive dysfunction to be found (Grande et al., 2017). In contrast, in patients with questionable cognitive impairment (QCI), cognitive function improved when they received anticholinergics, due to the therapeutic effects on comorbid pathologies (Swami et al., 2016). According to this, Fox et al. (2011a) have suggested that the anticholinergic action has to reach a critical threshold to produce cognitive effects. In addition, they postulate that there is a floor effect in the most advanced stages of neurodeterioration, in which cognition is not altered. The same group showed that the effect is higher in MMSE scores above 26 and is non-existent in scores below 21 (Fox et al., 2011b).

The combined use of anticholinergic drugs and AchI produces a higher rate of cognitive (Lu and Tune, 2003) and functional decline (Sink et al., 2008) with respect to those not taking anticholinergics at the same time, and this may induce an early discontinuation of the AchI treatment because its beneficial effect is being blocked by the anticholinergic drug due (Sverdrup Efjestad et al., 2017). However, higher cognitive decline of the combined treatment has been questioned in other studies (Bottiggi et al., 2007).

Despite the drawbacks of taking anticholinergic drugs, a novel approach that takes advantage of the peripheral anticholinergic action has been proposed in Alzheimer’s disease. A single-blind study with 41 moderate probable Alzheimer’s disease subjects showed that, after the co-administration of donepezil with the peripheral anticholinergic drug solifenacin 15 mg/day allowed the titration of donepezil up to 38 mg/day without notable adverse effects in 88% of patients. At 26 weeks, co-administration of solifenacin significantly improved the score of the ADAS-Cog, a widely used cognitive scale, versus baseline and versus the standard treatment with donepezil 10 mg/day. These results suggest that concomitant administration of AchI and a peripherally acting anticholinergic drug increases efficacy and tolerability of Alzheimer’s disease treatments (Chase et al., 2017). Nevertheless, the question remains on whether the addition of an AchI to a long-term treatment with anticholinergic drugs with central action that are not susceptible to modification could reduce the deleterious effects on cognition, functionality or mortality.

In brief, the higher the cognitive impairment the patients has, the more damaging is the effect of the anticolinergic effects of drugs, although this deleterious cognitive action may require exceeding a threshold to manifest. However in patients with advanced cognitive impairment, a ground effect may be found, and they will not present greater cognitive impairment when a certain level of deterioration is reached.

### Cognitive Outcomes in Schizophrenia

Cholinergic function in relation to cognition in schizophrenia has been recently studied in more detail. Patients with schizophrenia have twice the risk of developing dementia before the age of 80 than the general population (Tsoutsoulas et al., 2017). Cholinergic and dopaminergic systems, and the balance between them are essential for cognition, especially in people with schizophrenia (Ang et al., 2017). Unlike in affective disorders, in schizophrenia there is a low brain density of muscarinic receptors and patients usually have early cognitive dysfunction (Tsoutsoulas et al., 2017). Therefore, a light anticholinergic load can have a significant cognitive impact in schizophrenia, a finding not observed in schizoaffective disorder or bipolar disorder patients (Eum et al., 2017). In this line, data points toward a selective action of the M1 receptor in memory processes in schizophrenia. Thus, highly selective agonists of the M1 receptor would obtain better cognitive results than AchI, which are less specific (Vingerhoets et al., 2017).

Neuroimaging studies in patients with schizophrenia have found higher SAA related to the use of medications. Subjects with higher SAA have lower brain activation in the dorsolateral, anterior and medial prefrontal areas. These studies hypothesize that SAA interrupts modulatory prefrontal cholinergic transmission (Schreiber et al., 2018).

Ang et al. (2017) suggested that while cognitive impairment by anticholinergics in schizophrenia exists, it has a low effect size. A different study demonstrated a worse performance in speed processing and verbal memory related to the dose of antipsychotics and not to their anticholinergic action (Rehse et al., 2016). They also found that the worst performance was encountered if the antipsychotic drug had also a high anticholinergic action, except in patients receiving clozapine, a drug with clear anticholinergic effect. Considering these results, it is possible to hypothesize that NMDA agonism and M4 agonism can compensate for the anticholinergic action.

In patients with schizophrenia older than 50 years, a high anticholinergic load is associated with problems in spatial working and immediate memory and visuospatial ability. Deficits in attention, executive function and processing speed in schizophrenia patients may favor the presentation of a floor effect that masks the anticholinergic action. Anticholinergics may contribute to specific cognitive deficits in elders with schizophrenia that resemble those present in Alzheimer’s disease (Tsoutsoulas et al., 2017). On the other hand, there is strong evidence that if anticholinergic load is reduced in patients with schizophrenia, the performance of verbal memory and attention improves (Rehse et al., 2016).

At a functional level, the anticholinergic load associated with psychotropic drugs can alter the ability to participate and benefit from psychosocial treatments (O’Reilly et al., 2016).

In general, anticholinergic action of drugs in patients with schizophrenia are associated to worse cognitive outcomes. In fact, cognitive outcomes improve as the anticholinergic burden is reduced. However, it is suggested that other neurotransmitter systems may be involved in such cognitive impairment, since Clozapine shows better cognitive effects than others despite its intense anticholinergic action.

### Delirium

Delirium is a highly prevalent process in the elderly, especially in those suffering from dementia. Its onset has frequently been associated with anticholinergic drugs treatment (Collamati et al., 2016). The most accepted theory regarding delirium is the presence of diffuse imbalance of cerebral neurotransmission that implicates muscarinic receptors among others (Collamati et al., 2016). There is a biological plausibility about the association between anticholinergic drugs and delirium, but to date there are no conclusive results (Campbell et al., 2011; Wolters et al., 2015; Moorey et al., 2016). The variability of the scales used, age, presence of dementia, medical comorbidity, nutritional status and the possibility of reverse causation are among the most cited confounding factors (Collamati et al., 2016; Egberts et al., 2017).

### Emergence of Psychiatric Symptoms

Anticholinergic action has been related to the onset of psychiatric symptoms (mainly in the psychotic sphere) in elderly population with and without dementia, although the number of studies is still reduced.

In addition, it has been ruled out that this disposition to psychosis occurs indirectly due to cognitive function worsening (Cancelli et al., 2008). On the contrary, the procholinergic action of the AchI may be effective for controlling psychotic symptoms in dementia.

In a study in Alzheimer’s disease, the intake of anticholinergic drugs (except antipsychotics, to avoid reverse causation) increased the risk of psychosis, showing more risk for an increased anticholinergic load (Cancelli et al., 2008). This is in line with another study where the presence of SAA was associated with delusional ideation and diurnal rhythm disturbances (Hori et al., 2011). However, a different study (Dauphinot et al., 2017) found that the initial relationship between the anticholinergic load and the higher score in the Neuropsychiatric Inventory (NPI), a widely used scale to measure mental symptoms in neurodegenerative diseases, became non-significant after adjusting for confounding factors.

Hori et al. (2011) have also commented on the existence of the aforementioned “vicious cycle of anticholinergic activity in Alzheimer’s disease,” in which polypharmacy or the use of potent anticholinergics generates SAA and, in turn, this AA produces psychiatric symptoms that will be addressed with the prescription of new psychotropics with anticholinergic action.

In the face of risk factors such as old age, dementia, brain lesions, psychotic depression or bipolar disorder, cases of complex visual hallucinations related to the combination of serotonergic hyperactivity and cholinergic hypoactivity have been described. However, this a situation that is more frequent in patients on antidepressants, especially tricyclics (Cancelli et al., 2004).

### Functional Outcomes

Several studies have tried to correlate anticholinergic drugs with physical and functional deterioration. Treatment-related alterations would be due to their central action, but also to peripheral effects such as the difficulty with visual accommodation, tachycardia or gait problems.

The large majority of cross-sectional and longitudinal studies reviewed associate anticholinergic load measured with different instruments and loss of functionality. However, there are some studies that do not find a relationship between anticholinergic load and loss of functionality (Bostock et al., 2013; Sanders et al., 2017).

Mayer et al. (2017) found that the ARS and ADS scales had a high association with the Barthel index, a well-known scale to measure functionality. In a systematic review Wouters et al. (2017) concluded that the DBI was associated with changes in gait and basic and instrumental activities of daily life. The basic activities most affected by the anticholinergic action were bathing, grooming, dressing, transfers, mobility and stairs use (Lowry et al., 2011). A progressive decrease in global activity measured by accelerometers was also found (Clarke et al., 2018). A dose-response relationship between anticholinergic load and dysfunction has also been described. In fact, higher doses have a greater effect on people with more preserved previous functioning, and there is a floor effect in the most deteriorated patients (Sink et al., 2008).

The anticholinergic action affects the capacity for functional rehabilitation of both severe processes such as a stroke (Kose et al., 2018) and apparently minor injuries such as an ankle sprain (Kröger et al., 2019).

There are scarce studies regarding the influence of anticholinergic treatment on quality of life and they report contradictory data (Cossette et al., 2017; Wouters et al., 2017). Generally, there seems to be less psychological well-being in the patients taking anticholinergics, although it is not possible to exclude reverse causation because this association is not maintained when excluding patients diagnosed with depression (Teramura-Grönblad et al., 2011).

With regard to falls, it is estimated that up to 40% of community-dwelling elderly and even more of those living in institutions experience falls annually, with serious injury or mortality in 10% of cases (Marcum et al., 2015). In fact, falls are a frequent cause of hospitalization and institutionalization among old people (Zia et al., 2016). Anticholinergics intake has been associated with the presence of recurrent falls, although there is no definitive evidence in this regard (Lattanzio et al., 2018). It has been suggested that drugs with anticholinergic action may not be independently associated with the risk of falls and fractures (Fraser et al., 2014). The association between falls and anticholinergics could be related to dosage or duration of treatment, or to their non-anticholinergic effects such as sedation or orthostasis (Marcum et al., 2015), and mediated by the presence of changes in gait and balance (Zia et al., 2016). In fact, Wilson et al. (2011) found that only SSRI antidepressants, and no other anticholinergic drugs, have been associated with an increased risk of falls.

Thus, studies on the functional effects of anticholinergics have not shown unanimous results, but the large majority of them suggest a relationship between anticholinergic load and loss of functionality, with a threshold and a ground effect to produce these effects. With respect to falls, there seems to be a joint presentation of taking drugs with anticholinergic action and falls, although these falls could also be related to other patients’ factors and characteristics of drugs and not directly with anticholinergic action.

### Hospitalization and Outpatient Care

A systematic review found a relationship between anticholinergic load and hospitalization in five of the six included papers (Wouters et al., 2017). In institutionalized elderly, a moderate anticholinergic load increased the risk of hospitalization (Vetrano et al., 2016). A common finding is that hospitalization increases the percentage of patients who take anticholinergics, the number of anticholinergics per patient and the total anticholinergic load with respect to non-hospitalized patients due to reasons associated to hospitalization and/or treatment needs (Collamati et al., 2016). Additionally, a cohort study found an association between anticholinergic load and duration of hospital admission in both, patients with and without dementia (Gnjidic et al., 2014); however, this association was not found in other studies (Lowry et al., 2011; Egberts et al., 2017).

A higher risk of institutionalization in nursing homes has been found to be proportional to the anticholinergic load regardless of the presence of delirium and after adjustment for comorbid conditions (Egberts et al., 2017). In addition, a greater anticholinergic load has been associated with a greater number of outpatient visits and visits to the emergency room (Campbell et al., 2016).

So, in general, both institutionalization and hospitalization appear temporarily associated with the use of anticholinergic drugs. However, the relationship between hospitalization and its characteristics and the consumption of anticholinergic drugs before and after hospitalization could be indirect and due to one of the other multiple factors involved.

### Mortality

Cardiovascular anticholinergic effects such as tachyarrhythmia, syncope, ischemia or neurological effects such as epileptic seizures, hallucinations, and delirium may increase mortality (Vetrano et al., 2016; Wouters et al., 2017).

There are no conclusive results about greater mortality in hospitalized elderly patients taking anticholinergics. However, mortality has been associated with anticholinergic use in the presence of hyponatremia (Lowry et al., 2011) or delirium (Mangoni et al., 2013), especially in the presence of cognitive impairment (Lattanzio et al., 2018), even 1 year after discharge.

Anticholinergics have also been found to be associated with higher mortality in institutionalized elderly, especially in those with coronary disease (Vetrano et al., 2016) due to the greater vulnerability to the proarrhythmic effects of these drugs. A longitudinal study found a dose-dependent association between taking anticholinergics and the risk of stroke and death in elderly people with Alzheimer’s disease, mixed dementia and vascular dementia (Tan et al., 2018) which would parallel to the well-known risk of antipsychotics. Nevertheless, this risk does not seem to be directly associated to the possible anticholinergic effect of antipsychotics, since it is very different among the different drugs (Maust et al., 2015).

## Consequences of Discontinuation of Anticholinergic Drugs

Deprescription has the potential to reduce or eliminate unnecessary or potentially inadequate drugs, reduce polypharmacy and improve different health variables. Eliminating or reducing the anticholinergic load can improve cognition, adverse effects and quality of life (Lupu et al., 2017). For example, in patients with schizophrenia younger than 50 years, the discontinuation of anticholinergics improved the results in the Wisconsin Card Sorting Test, a measurement of executive dysfunction (Sathienluckana et al., 2018).

However, some experts suggest that there is little evidence regarding the reversibility of anticholinergic cognitive effects through discontinuation and question the safety of this measure. Different studies have proposed conflicting hypotheses regarding the reversibility of cognitive effects after discontinuation. Several of them point to a long-term irreversibility, although it is unknown whether a temporary threshold for such irreversibility exists. In a systematic review including four studies (two randomized controlled trials and two prospective cohort studies), only the cohort studies showed cognitive improvement after anticholinergic drugs discontinuation (Salahudeen et al., 2014). In contrast, a different prospective cohort study conducted by Gray et al. (2015) did not find attenuation of the risk of dementia or Alzheimer’s disease with discontinuation. A sub-analysis of a single-blind randomized trial that did not find cognitive improvement showed that the subgroup of patients in whom the anticholinergic load could be eliminated had a greater improvement in immediate memory. This finding suggests that the cognitive improvement could be dose- dependent or could require the total discontinuation of anticholinergics (Kersten et al., 2013). At the same time, it has also been suggested that a long anticholinergic-free period is required for cognitive improvement.

Nonetheless, discontinuation is not always possible and clinical reasons for not carrying out the discontinuation should always be considered (Lupu et al., 2017). For example, in some patients with schizophrenia, withdrawing benztropine may clearly worsen extrapyramidal symptoms related to antipsychotic medication, and therefore maintenance of the treatment may be required.

Thus, the discontinuation of anticholinergic drugs should be an objective due to their adverse effects. However, this objective should be flexible because the cognitive effects may no longer be reversible or there is an evident risk of serious worsening of an underlying disease.

## Discussion and Recommendations for Clinical Practice

Geriatric prescription of anticholinergic drugs cannot always be avoided in clinical practice. If prescription is necessary, priority should be given to drugs with a mild anticholinergic load (Bishara et al., 2017) and those with high affinity for their site of action (Collamati et al., 2016). In addition, possible medication management problems should be addressed in view of the increased anticholinergic load. In fact, this has been related to adherence problems especially in male patients (Boccardi et al., 2017).

When an anticholinergic drug is prescribed, cognitive function and comorbidities sensitive to anticholinergic effects such as benign prostatic hypertrophy, constipation, gait problems, among others, should be assessed, together with the already existing avoidable or inevitable anticholinergic load caused by concomitant treatments.

Given the potential risk of irreversible cognitive effects in prolonged treatments, the efficacy and risks of anticholinergic drugs should be re-evaluated when treatment periods longer than 3 months are considered (Cai et al., 2013; Wu et al., 2017).

Abrupt discontinuation is not recommended due to the risk of a cholinergic rebound, i.e., symptoms of agitation, diarrhea, vomiting, lacrimation, tachycardia, insomnia and movement disorders after withdrawal (Lupu et al., 2017). Patients should be informed of potential risks of discontinuation, and participate in therapeutic decisions (Bishara et al., 2017).

Iatrogenesis caused by anticholinergic drugs could be reduced with the availability of the opinion of a clinical pharmacologist, the use of anticholinergic load calculators, tools such as the “Pocket Reference Card for Medicines with Anticholinergic Activity” or future software that generates therapeutic alternatives to specific clinical problems (Salahudeen and Nishtala, 2016).

The following are practical recommendations oriented to different clinical conditions.

- ‘Dementias, including neuropsychiatric symptoms in dementia.’ The anticholinergic risk is higher in dementias with cholinergic deficit such as Alzheimer’s disease, Lewy body dementia and Parkinson’s dementia. When the use of AchI produces procholinergic adverse effects, low doses should be used, titration should be slower, or their use should be abandoned, instead of prescribing anticholinergic drugs to alleviate these side effects. In a patient presenting symptoms of dementia, a discontinuation of anticholinergics that results in cognitive improvement should question the neurodegenerative origin of the problem, or at least consider that the medication has acted as an AA insert, which means that the cholinergic system is altered but is functional without external interference. On the contrary, the absence of improvement after the discontinuation of the anticholinergic drug would favor the diagnosis of dementia. If the use of antipsychotics is necessary, those with less anticholinergic action should be prioritized. In fact, in Europe, Risperidone is the only atypical antipsychotic licensed drug for the treatment of neuropsychiatric symptoms in dementia. The anticholinergic burden attributed to Risperidone varies in the different medication lists. There are other antipsychotic drugs, such as Aripiprazole, considered less anticholinergic. However, nowadays the main focus on the safety of the use of antipsychotics in dementia is oriented to the greater risks of cerebrovascular events and death. These effects are serious, and the reason why antipsychotics in dementia should only be prescribed in the short term and only after having tried non-pharmacological options.
- Schizophrenia. Antipsychotics and anticholinergic correctors used for extrapyramidal side effects of antipsychotics are negatively associated with cognition (Ziad et al., 2018). The cognitive effects of anticholinergics should be closely monitored in patients with greater baseline cognitive impairment. Except in the acute phases, the minimum doses of antipsychotic drugs should be used to avoid or reduce the additional use of anticholinergic correctors for extrapyramidal side effects, which must be avoided as much as possible in the elderly (Rehse et al., 2016; Lupu et al., 2017). This should be considered in all cases but in acute or tardive dystonias that respond to anticholinergics the withdrawal may not be possible (Lupu et al., 2017). Since schizophrenia is a chronic disease, the anticholinergic load in patients suffering from it can increase with treatment time and by the treatments prescribed for other comorbidities (Tsoutsoulas et al., 2017).
- Acute hallucinatory episode. The action of antidepressants and other serotonergic and/or anticholinergic drugs should be assessed in order to withdraw them before considering the prescription of antipsychotics (Cancelli et al., 2004).
- Depression. Given the risk of progression to dementia in an insufficiently treated depression, the first measure is to try to achieve remission. In a limited number of cases, patients with prolonged treatment with tricyclic antidepressants could be harmed by switching to SSRIs if their effectiveness on depression is lower (Lupu et al., 2017). Among antidepressants, amitriptyline has a high anticholinergic load already at a dose of 25 mg/day, nortriptyline has a moderate load at 50 mg/day and paroxetine has also a moderate load at 30 mg/day (Chew et al., 2008).
- Anxiety. Benzodiazepines may cause cognitive impairment due to several different mechanisms, including anticholinergic effects when prescribed at high doses. Lorazepam has a short action and is low fat-soluble, causing less cognitive effects than other benzodiazepines (Bishara et al., 2017).
- Parkinson’s disease. Anticholinergic drugs should be discontinued if cognitive dysfunction occurs and should no longer be reintroduced (Nishiyama et al., 1998).
- Cardiovascular diseases. Drugs with anticholinergic action such as warfarin or digoxin are essential for the prevention of cardiovascular and cerebrovascular events and do not always have adequate alternatives (Pasina et al., 2019). Therefore, their use should be prioritized over other drugs with anticholinergic action that may be necessary to prescribe. Additionally, some drugs with cardiovascular action can exert compensatory protective effects on cognitive dysfunction (Richardson et al., 2018).
- Urinary incontinence/bladder dysfunction. Non-pharmacological alternatives such as changes in lifestyle, bladder retraining and physiotherapy should be considered first (Araklitis et al., 2017). If anticholinergics are prescribed and clearly improve symptoms and quality of life, discontinuation may not be appropriate. Among the drugs of its class, tolterodine and oxybutynin are the urinary incontinence drugs with the highest anticholinergic load (Araklitis et al., 2017). Oxybutynin has the highest blood-brain barrier permeability and trospium the lowest. Darifenacin has the lowest risk of interaction with AchI (Sverdrup Efjestad et al., 2017). In multiple sclerosis, alternatives such as the use of botulinum toxin injections or mirabegron should be considered (Morrow et al., 2018).

## Conclusion

Anticholinergic agents are clearly useful for the treatment of several processes that involve different organs and systems, such as cardiovascular, respiratory and CNS s, among others. In the elderly, side effects are more commonly found. They can manifest acutely, such as constipation, reversible cognitive dysfunction or delirium, or after a long exposure, such as an increased risk of developing dementia, hospitalization or increased mortality.

The risk-benefit ratio should always be assessed when prescribing a drug with anticholinergic effects. It is important to balance the risk of using them and the risk of not using them and to evaluate pharmacological and non-pharmacological alternatives. Assessment must always include medical comorbidities and other medications that the patient is receiving. To sum up, the research line that differentiates adequately and inadequately prescribed anticholinergic drugs seems very promising and more studies should be done considering the knowledge gaps reviewed.

## Author Contributions

JL-Á and LA-O designed the project. JL-Á managed the literature search and wrote the first draft of the manuscript. LA-O and JS-L-J revised the drafts and supervised the English translation. JL-Á, LA-O, and JS-L-J reviewed and contributed equally to the final version of the manuscript.

## Conflict of Interest

The authors declare that the research was conducted in the absence of any commercial or financial relationships that could be construed as a potential conflict of interest.
